# The Common Single Cause of Chronic Multi-Hormonal Resistance in Oxidative Stress

**DOI:** 10.3390/antiox12010075

**Published:** 2022-12-29

**Authors:** István Wittmann

**Affiliations:** 2nd Department of Medicine and Nephrology-Diabetes Centre, University of Pécs Medical School, H-7624 Pécs, Hungary; wittmann.istvan@pte.hu; Tel.: +36-72-536-050

**Keywords:** chronic hormonal resistance, diabetes mellitus, glucagon-like protein-1 receptor agonist, insulin receptor substrate, meta-tyrosine, ortho-tyrosine, oxidative stress, tyrosine phosphorylation

## Abstract

In diseases with concomitant oxidative stress, chronic multi-hormonal resistances could be detected. The most conspicuous component of these resistances is insulin resistance, but also leptin, erythropoietin, acetylcholine, triiodothyronine and glucagon-like peptide-1 resistances also occur. On the other hand, in oxidative stress, abnormal tyrosines, for instance, meta- and ortho-tyrosine are also produced and incorporated into the proteins through the translational process. In case these modified proteins are components of the intracellular signalling pathways, a hormonal resistance may develop. The above-mentioned hormones, owning overlapping signalling pathways at the insulin receptor substrate, develop an abnormal tyrosine phosphorylation dependent chronic multi-hormonal resistance. A few weeks free of oxidative stress or the use of antioxidant therapy are required to provide a return from this resistance, which return may be further supported by the supplementation of physiological para-tyrosine and by the add-on therapy of a pharmacological dose of glucagon-like peptide-1 receptor agonist, which is able to bypass the critical insulin receptor substrate signalling.

## 1. Introduction

In chronic diseases such as obesity, prediabetes and diabetes mellitus, a chronic, inappropriately high energy intake is the major driver of the changes of gut microbiome, of which metabolites pass the gut wall and this leads to phenotype changes of abdominal fat cells resulting in the increase of their secretory function manifesting in the excretion of cytokines (e.g., TNF-α, IL-6) [1]. This higher circulating level of cytokines and abnormalities of immune cells constitute the background of the so-called systemic subclinical inflammation targeting all cells of the body causing finally, at the subcellular level, oxidative stress, which is a characteristic feature of such obese, prediabetic and diabetic patients [2]. Their chronic insulin, incretin and leptin resistances is also well described [3,4] and other resistances are assumed.

Not only the subclinical but also an overt inflammation due to the infections (e.g., in SARS-CoV-2 [5]) or autoimmune diseases (e.g., in lupus [6]) cause an oxidative stress and metabolic abnormalities in the cells. Moreover, in acute severe infections, e.g., in septic state, an oxidative stress could be verified [7] with an elevation of glucose level as a consequence of the disturbed insulin signalling leading to insulin resistance [8,9], however, other hormonal resistances are usually not searched for.

Hormonal resistance in subclinical inflammation is rather chronic in its nature, but it may also have some acute components, and in the clinically overt systemic inflammations the sharing of acute and chronic components depends on the duration of the inflammation. Acute and chronic inflammation lead to acute and chronic hormonal resistances. The question arises whether acute and chronic hormonal resistances differ in their clinical appearance and by their pathophysiology.

The aim of this review is to summarize the available data and evidences regarding the possible role of oxidative stress in the development of chronic multi-hormonal resistances.

## 2. Intracellular Signaling of Insulin, Leptin, Acetylcholine, Glucagone-Like Peptide-1, Erythropoietin and Triiodothyronine

### 2.1. Insulin Signaling

In spite of the discovery of insulin a long time, 100 years ago, still the exact intracellular effect of it is not completely understood. Insulin receptor, after binding of insulin, being activated by autophosphorylations at its tyrosine residues initiates a phosphorylating cascade of many intracellular signalling proteins, as for example the insulin receptor substrate (IRS) family at their tyrosine residues. In this cascade, involving protein-protein interactions and phosphorylation of phosphoinositide as well, Akt is also activated. For the full intracellular metabolic action of insulin, tyrosine phosphorylation requires selective activation of the protein kinase C (PKC) family as well. Protein tyrosine phosphatase enzymes rapidly dephosphorylate the crucial phospho-tyrosines regulating the system and making an on/off response possible. Defects of both tyrosine phosphorylation and dephosphorylation are responsible for insulin resistance, although, abnormalities of activation of different PKC isoforms also play a role, however, their selective inductions are not completely independent from the tyrosine cascade [10], but glucometabolic effect of insulin signalling is not directly linked to JAK2-activation.

### 2.2. Triiodothyronine Signaling

In leptin receptor-deficient db/db rats has proved that triiodothyronine exerts an antidiabetic effect. In the same study, in an in vitro experiment, in 3T3-L1 adipocytes, triiodothyronine potentiated insulin induced activating tyrosine phosphorylation of IRS-1 [11], but regarding JAK2 involvement no data are available.

### 2.3. Leptin Signaling

Six leptin receptor isoforms exist (LepR-a-f), of which LepR-e is a soluble form. The others have a transmembrane domain with three crucial tyrosine residues, which are phosphorylated by Janus kinase-2 (JAK2) this step being a *sine qua non* of leptin signalling. Tyrosine phosphorylated leptin receptor activates signal transducer and activator of transcription-3 and 5 (STAT3/STAT5), and IRS [12,13,14]. Obesity induced chronic inflammation leads to the inhibition of leptin signalling and accordingly to leptin resistance [15].

### 2.4. Acetylcholine Signaling

Muscarinic receptorial activation by acetylcholine in endothelial cells involves similar pathways as that of leptin. A JAK2-activated tyrosine phosphorylation of IRS-1-Akt-endothelial nitric oxide synthase enzyme (eNOS) pathway was described for acetylcholine as well [16]. This is also overlapping at IRS-1 and Akt with the previously mentioned insulin signalling. Moreover, in these experiments, obesity was related to both insulin and acetylcholine signalling defects, i.e., obesity caused a resistance to both hormones [16].

### 2.5. Erythropoietin Signaling

Erythropoietin receptor dimer, with the associated JAK2 kinase, after binding of erythropoietin is be activated by a transphosphorylation process. JAK2 phosphorylates own and erythropoietin receptor tyrosine residues. STAT5 is the next signalling protein which is phosphorylated by the activated erythropoietin receptor-JAK2 complex. Activated STAT5 is responsible for the proliferation of erythroblasts [17].

In skeletal muscle, another signalling pathway was also described, referring to the possible activation of JAK2-IRS-1-PI3K-Akt-eNOS cascade [18].

### 2.6. Glucagone-Like Peptide-1 (GLP-1) Signaling

Insulin secretion promoting effect of GLP-1 in the beta-cells is mainly mediated by an adenylyl cyclase-cAMP pathway. On the other hand, there is an alternate, insulin-related effect potentiating GLP-1 signalling as well. This is associated with GLP-1 induced betacellulin production, which activates the antiapoptotic EGFR-Grb2-Gab1-PI3K-PDK-Akt signalling [19]. Gab1 and IRS are structurally similar, with essential tyrosine phosphorylations [20]. The GLP-1-related beta-cell mass retention is a crucial process in saving insulin response in type 2 diabetes.

### 2.7. Common and Distinct Signaling Pathways

**Figure 1:**
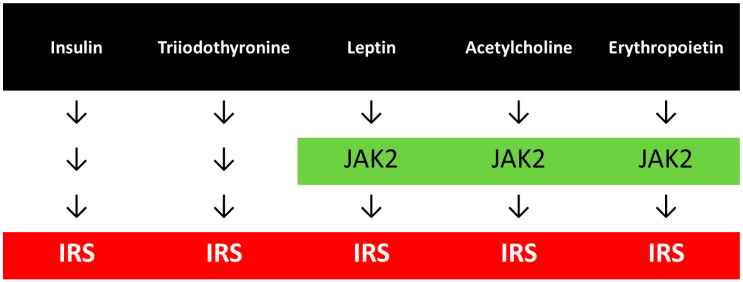
Hormonal signallings with common pathways.

While insulin and triiodothyronine provide direct activation of the IRS, other hormones such as leptin, acetylcholine and erythropoietin, indirectly, through the stimulation of JAK2 exert the same IRS signalling (Figure 1), and other hormones may share this pathway as well. GLP-1 signalling is more complicated resulting in an activation of the Akt. The common criteria for the normal transduction of these hormonal signalings are the well-working essential tyrosine phosphorylation-dephosphorylation processes.

### 2.8. Acute Redox Regulation of Hormonal Signaling and Metabolism

Acute stressors, as metabolic factors, e.g., hyperglycaemia, elevated plasma level of free fatty acid (FFA) or advanced glycation end-products (AGE), inflammatory mediators, such as cytokines (TNF-α, IL-6), hormonal changes, as an enhanced secretion of renin, angiotensin II, aldosterone, catecholamine, or hypoxia, as in case of obstructive apnoea syndrome and many others, evoke acute intracellular oxidative stress, which results in acute alterations of the redox regulation of hormonal and metabolic signalling [21]. Redox regulation was already suggested in the early 20th century by A. Szent-Györgyi, which redox reactions are able to exert an effect on metabolism and extracellular signal-regulated kinase 1/2 (ERK1/2), Jun N-terminal kinase 1/2 (JNK1/2), nuclear factor κB (NFκB) and protein tyrosine phosphatase-1B (PTP-1B) [22,23].

A network of these acute alterations is well described [24], however other players could also be supposed in the background of insulin resistance.

All of these network alterations lead to an acute modulation of intracellular insulin signalling, resulting in the changes of insulin receptor substrate-1 (IRS-1) phosphorylation, which is assumed to be a key element of insulin resistance. In an insulin resistant state, instead of the physiological tyrosine phosphorylation, serine phosphorylation was described [25].

However, in hormonal resistant state due to subclinical inflammation the chronic alterations seem to drive the changes of signalling which are not able to be eliminated by acute antioxidant therapy.

### 2.9. The Oxidative Stress Memory

Metabolic memory of the body is well known and also clinically proved, which orients the therapeutic management of metabolically ill patients, as e.g., in the case of diabetes mellitus, but vascular memory was also hypothesized [26]. Early metabolic control of diabetic patients was suggested to be able to avoid cardiovascular complications due to the metabolic memory.

Oxidative stress being a major determinant of both metabolic and vascular diseases, oxidative stress memory may also be assumed. Moreover, insulin resistance of the immune cells, such as macrophages due to the oxidative stress [21] may change their function leading to immunological memory of oxidative stress at the same time.

What molecular basis could be hypothesized to be in background of oxidative stress memory? Either DNA or protein damages may explain its legacy. Changes which are reversible may have a protein-related origin, whereas those that are not reversible are rather DNA based.

Thus, to make a differentiation between the DNA and protein-related origin of chronic multi-hormonal resistances, we have to reflect on the reversibility of these states.

### 2.10. The Breakthrough Phenomenon

In the clinical routine a break-through treatment is used in recently diagnosed type 2 diabetic patients, which means a 1 to 4 weeks intensive insulin therapy, introducing either human or analogue insulins, or even insulin pump management. During this therapy a near normalization of glycaemia by insulin leads to the remission of diabetes mellitus, constituting an improvement of own insulin secretion and a reverse of insulin resistance, both of which make stopping of insulin regimen possible. This remission period could be prolonged even to several years without insulin therapy [27,28].

On the other hand, an implementation of insulin for a break-through therapy in type 2 diabetic patients may correct the resistance of glucagon-like peptide-1 (GLP-1) and of the glucose dependent insulinotropic peptide (GIP) alike, which are the other hormonal types of resistance characteristic of this diabetic population [29]. Interestingly, *vice versa*, a pharmacological dose of a dual activator of GLP-1 and GIP receptors, tirzepatide, ameliorates insulin resistance [30].

These clinical data suggest that resistances of insulin, GLP-1 and GIP are interactive and reversible, raising the probability of the protein-related nature of the resistances of these hormones.

### 2.11. Consequences of Oxidative Stress at the Protein and Amino Acid Level

There are many modifications of the proteins due to an oxidative stress. Different oxidizing species induce various protein modifications. Hydroxyl-, alkoxyl-, peroxyl-, superoxide-, carbonate- and nitrogen dioxide radical have distinct targets on the protein, and non-radical oxidants such as peroxynitrous acid, hydrogen peroxide and others may damage also proteins. Among others, fragmentation, carbonylation, dimerization, hydroxylation and disulfide, sulfonic and sulfinic acid, sulfoxide, sulfon, kynurenine, alcohol, hydroperoxide formation could be detected [31]. These posttranslational modifications of the protein structure lead to their functional changes. Cells are especially sensitive to protein modification if they change not only the structural but also the signalling proteins causing a disruption of the regulation of the metabolic or other functions of the cell.

Reactive oxygen species may attack not only proteins but also amino acids. Hydroxyl free radical is able to produce aromatic ring hydroxylation, for instance in the case of phenylalanine. The end-products of these reactions are para-, meta- and ortho-tyrosine (p-Tyr, m-Tyr, o-Tyr).

### 2.12. Sources of Normal (Para) and Abnormal (Meta and Ortho) Tyrosines

p-Tyr is a physiological, semi-essential amino acid with three sources for the human body (Table 1). Partially it comes from the food, and it is also produced enzymatically in the liver and kidney. The normal plasma level of p-Tyr in healthy persons could be found to be 55.96 μmol/L in our examination [32]. Compared to the absorbed and enzymatically produced p-Tyr, a small amount of p-Tyr is formed non-enzymatically by the hydroxyl free radical attack of phenylalanine, which could be in the low nanomolar range [32]. Abnormal m-Tyr and o-Tyr are formed in the human body due to the hydroxyl free radical attack of the phenylalanine (plasma level of o-Tyr proved to be 22 nanomol/L in healthy persons [32]), but it is also produced enzymatically in plants (Table 1).

There are no human data available regarding the absorbed amount of abnormal amino acids through the gastrointestinal tract from the plant-based foods, but, according to our data, these abnormal amino acids are rapidly transported by human cells [21], and o-Tyr is absorbed from the gastrointestinal tract of rats (Table 1 [33]). Meat damaged by oxidative stress and plants accumulating m-Tyr may serve as sources of abnormal tyrosines for the absorption from the gastro-intestinal tract.

### 2.13. Overproduction and Reduced Elimination of Abnormal Tyrosines

As mentioned in the Introduction, in obesity, prediabetes and diabetes cytokines produced by the abdominal fat cells induce the so-called systemic subclinical inflammation.

In systemic subclinical inflammation (Table 2), in diabetes mellitus we detected a significant increase of urinary excretion of o-Tyr (urinary o-Tyr/urinary creatinine, median and interquartile range, in μmol/mmol): in healthy, 0.034 (0.0001–0.035), in diabetes, 0.291 (0.103–0.330), which is almost 10-fold higher [32].

In dyslipidaemia, no data of abnormal tyrosines are available for humans, but in animal experiments, in cholesterol fed rats we could detect a significant increase of m-Tyr in the thoracic aortic wall [34].

In septic patients, plasma p-Tyr level significantly decreased on the first day of sepsis, and the urinary o-Tyr excretion increased in some patients above 2 μmol/mmol [35].

In chronic kidney disease (CKD), the patients’ (eGFR, 35 mL/min) urinary o-Tyr/creatinine ratio proved to be 0.175 μmol/mmol, which is 5-fold higher compared to the values of healthy individuals. In patients with diabetes and CKD a value of 0.479 μmol/mmol could be verified, which amounts to a 14-fold higher value [32].

In patients with end-stage kidney disease (ESKD) on haemodialysis a significant decrease of p-Tyr and a significant (more than 20-fold) elevation of o-Tyr were detected [36].

Importantly, in the human body, the majority of the enzymatically produced export p-Tyr for the other organs originates from the kidney, and in kidney damage (acutely e.g., in sepsis and chronically in CKD) its plasma level drops [37]. 

In the cells, the decrease of the physiological p-Tyr and increase of the availability of abnormal tyrosines and the resulting reduction of p-Tyr/m-Tyr+o-Tyr ratio could be the worst combination regarding the translation of normal proteins.

### 2.14. Translational Incorporation of Abnormal Tyrosines into the Proteins

Incorporation of oxidized amino acids into the proteins of mouse macrophage cell line was examined using m-Tyr and L-3,4-dihydroxyphenylalanine (DOPA). It was verified that the presence of these amino acids in the proteins is a result of protein synthesis [38]. In another study, protein syntheses was studied in an Escherichia coli cell-free system and proved that more than 90% of replacement of tyrosine by DOPA was achieved [39]. Using also an *E. coli* cell-free protein synthesis system incorporation of ring-substituted phenylalanines and tyrosines was investigated and their misincorporation into the proteins was detected [40].

Interestingly, some plants enzymatically produce and accumulate m-Tyr in their root, and release it to the soil inhibiting the development of its surrounding plants. These surrounding plants misincorporate m-Tyr into their proteins, which leads to damages of their cells [41].

Our group proved that p-Tyr, m-Tyr and o-Tyr were rapidly transported into the 3T3-L1 adipocytes, independently of the medium glucose concentration and the presence or absence of insulin [21]. We also demonstrated that m-Tyr and o-Tyr were incorporated into the cellular proteins of adipocytes, HEK cells, podocytes, macrophages, erythroblasts and endothelial cells [21,33,42]. It was also verified that in the cases of adipocytes and erythroblasts p-Tyr competitively prevented abnormal tyrosine incorporation [21,42].

Degradation of proteins modified by DOPA or m-Tyr was investigated in a macrophage cell line and a lower degradation was described in the case of DOPA but not for m-Tyr or o-Tyr containing proteins. This study also noted that the machinery of degradation was not affected by the culturing of the cell with oxidized amino acids, thus the normal protein degradation remained intact [38].

## 3. Abnormal Tyrosine Content of Proteins as a Common Cause of Chronic Multi-Hormonal Resistance

### 3.1. Insulin and GLP-1 Resistance

In in vitro experiments, 3T3-L1 adipocytes are frequently used for the study of insulin resistance [43]. In our study, insulin induced glucose uptake of these cells was decreased grown on high glucose concentration (25 mmol/L) or on m-Tyr or o-Tyr containing culture media [21]. Inhibitory effect of abnormal tyrosines was abolished by the excess of normal p-Tyr. The signalling of insulin was also studied and an activating tyrosine phosphorylation of IRS-1 and the downstream Akt activation were investigated, which were diminished in the cells grown in the medium containing m- or o-Tyr. High glucose or abnormal tyrosines were not present in the media at the insulin stimulation, only during the culturing of the cells [21].

These investigations were repeated in HEK cells, podocytes and macrophages with similar results. Interestingly, non-stimulated, basal Akt phosphorylation was significantly higher in adipocytes, HEK cells and in podocytes, just as the non-stimulated IRS-1 phosphorylation in macrophages. This suggested a set point shift, which resulted in the resistance to stimulation of insulin [21].

In our other examinations we proved that a chemically synthesized, non-phosphorylated, substrate site of insulin receptor, a region of human IRS-1 (region 626–639) was phosphorylated by insulin receptor very weakly if the tyrosine was m-Tyr or o-Tyr, and excellently in case it was p-Tyr, and dephosphorylation of the phosphorylated polypeptide was almost impossible if the phosphorylated tyrosines were m-Tyr or o-Tyr, while phosphorylated p-Tyr was rapidly dephosphorylated by protein tyrosine phosphates 1B enzyme [21].

Moreover, binding of the synthesized phospho-polypeptide to the downstream signalling protein, to PI3K was inhibited if the polypeptide contained phosphorylated m-Tyr or o-Tyr [21].

In an in vivo experiment we fed rats using o-Tyr and after 4 weeks we detected an elevated o-Tyr content of the vascular wall of the femoral artery accompanied by the diminishing of vascular relaxation to insulin. In the same study, 8 days of o-Tyr supplementation of the culture media of endothelial cells resulted in a significant increase of the o-Tyr content of proteins of the cells and in a decrease of the activating phosphorylation of the eNOS enzyme [33].

In another in vivo study, in cholesterol fed rats, we measured an increased level of m-Tyr in the thoracic aorta together with its decreased relaxation to insulin and to liraglutide, an agonist of GLP-1 receptor, which damage was reversed by p-Tyr supplementation [34].

In an in vivo human study of septic patients we verified that the independent predictors of the damage of carbohydrate metabolism were serum phenylalanine (Phe), p-Tyr/Phe, o-Tyr/Phe, as well as urinary excretion of m-Tyr, m-Tyr/p-Tyr, o-Tyr/p-Tyr and m-Tyr+o-Tyr/p-Tyr [44]. In yet another human study of our workgroup, in diabetic patients, effects of resveratrol were investigated and it was shown that resveratrol improves sensitivity of insulin and activates Akt, accompanied with a decrease of urinary excretion of o-Tyr [45].

### 3.2. Triiodothyronine Resistance

In a human, in vivo study we demonstrated that metformin reduces resistance of triiodothyronine and insulin in euthyroid, recently diagnosed type 2 diabetic patients. As a part of this examination, in an in vitro experiment using Western blot, we proved that in HEK-cells activating phosphorylation of Akt due to triiodothyronine was abolished by high glucose concentration [46]. Unfortunately, in these experiments the role of abnormal tyrosines was not directly examined, however, one could hypothesize that the high level of glucose evoked glucotoxicity and the consequently induced oxidative stress is manifested in the same pathogenesis as in the case of insulin resistance.

### 3.3. Acetylcholine Resistance

Acetylcholine induced vasodilation is a well-known model for the study of the health of vasculature. We described a proximal to distal decrease of the o-Tyr content of the wall of the macrovasculature as follows: thoracic aorta > abdominal aorta > femoral artery [47]. According to the decrease of o-Tr gradient-, insulin- and acetylcholine-induced an increase of vasodilation to the periphery [48].

### 3.4. Erythropoietin Resistance

In about 15% of dialyzed ESKD patients an erythropoietin resistance could be detected [49], which frequently leads to the need of a high dose of erythropoiesis stimulating agents (ESA). As was shown in Figure 1, erythropoietin signalling results in tyrosine phosphorylation of JAK2, erythropoietin receptor and IRS, and in a downstream step in an activating phosphorylation of STAT5.

Knowing these multiple phosphorylations of tyrosine residues of erythropoietin signalling, we studied in an in vitro erythroblast model the effect of m-Tyr and o-Tyr on the STAT5 phosphorylation. We noticed that a three-day long incubation by abnormal tyrosines almost completely blocked the signal transduction [42]. On the other hand, m-Tyr and o-Tyr decreased the erythropoietin induced proliferation of erythroblast cells. We concluded that this pathomechanism could be responsible for the chronic erythropoietin resistance.

In an in vivo human study, observing ESA-dose and the erythropoietin resistance index (ERI) of dialyzed patients, including most of the risk factors, the independent predictor proved to be the plasma o-Tyr/p-Tyr ratio [36]. These results were independent of the ERI formula used (involving either haemoglobin, or haematocrit or red blood cell number).

### 3.5. Effect of Antioxidants on Multi-Hormonal Resistance

A large number of clinical investigations were dealing with antioxidants and insulin resistance concluding that an antioxidant treatment in diabetic or obese patients could serve as a valuable management method for the reduction of insulin resistance using either α-lipoic acid [50,51,52] or natural antioxidants, such as resveratrol [53], but these data are not accepted as high level evidences in guidelines, since big randomized, controlled trials are still lacking. In these clinical investigations dealing with antioxidants and insulin resistance, duration of the treatment by antioxidants was usually a few weeks or months long, supporting again that an acute antioxidant treatment fail to improve insulin resistance and that insulin resistance is reversible.

In another chronic clinical study, in a group of dialyzed diabetic patients, the effect of α-lipoic acid was investigated and found to be effective in decreasing haemoglobin A_1c_ and the resistance of erythropoietin as well [54].

In chronic haemodialysis, using vitamin E-coated membranes improves anaemia management by decreasing erythropoietin resistance [55].

A high (pharmacological) dose of some hormones may influence the resistance of another hormone. Indeed, in animal experiments, a treatment by GLP-1 receptor agonist, liraglutide, induced in obese mice an improvement of antioxidant defence and also a decrease of leptin resistance was detected [56]. Additionally, in the same model erythropoietin alleviated insulin resistance [57]. These results are in agreement with clinical examinations explaining the beneficial effect of a break-through therapy by insulin on GLP-1 resistance and effect of GLP-1 and GIP on insulin resistance as pointed out above.

There are no data available regarding a favourable effect of antioxidant treatment on acetylcholine or triiodothyronine resistance, however, according to our results, metformin was unable to prevent high glucose induced resistance of triiodothyronine in a cellular model [46].

## 4. Conclusions

**Figure 2:**
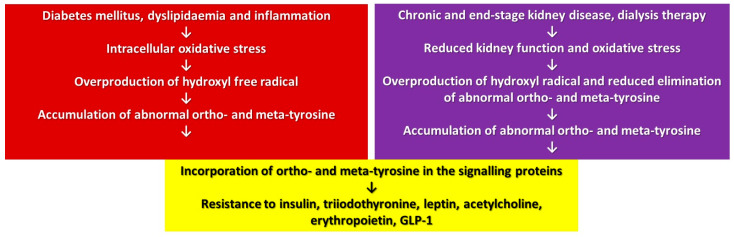
Mechanism of development of multi-hormonal resistance in oxidative stress.

A posttranslational modification of proteins may change and, in some cases, derange their function. In our approach, a translational modification of proteins has a similar effect due to the overproduction or reduced elimination of abnormal tyrosines leading to their accumulation in the cellular proteins (Figure 2). This accumulation of abnormal tyrosines results in pronounced cellular changes if the signalling proteins are affected, since its consequences will be augmented by the signalling cascade producing a multi-hormonal resistance of the cells due to the overlapping signalling pathways of these hormones (Figure 2). The present review summarized the actually available data mainly related to obesity, diabetes, CKD and sepsis, but one may suppose that, in the case of any other origin of the oxidative stress, similar processes could take place.

## 5. Future Perspectives

**Figure 3:**
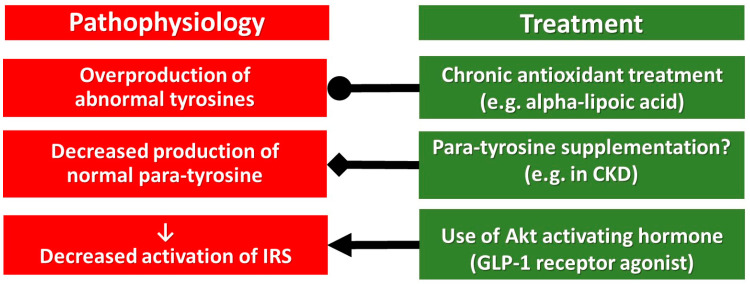
Pharmacological treatment of the pathophysiological targets of chronic multi-hormonal resistance. Abbreviations, ●, inhibition; ◆, supplementation; ⟵, activation.

Conclusions drawn above are based mainly on non-human experiments, but the preliminary clinical examinations may suggest some therapeutical consequences as well (Figure 3).

Usage of antioxidants in the clinical routine is debated, but alpha-lipoic acid is administered in everyday practice for the treatment of diabetic neuropathy. It is effective and is without severe side effects, only a few hypoglycaemic episodes are detected. Its favourable hormone sensitizing side effects have already been mentioned above, but further randomized, controlled clinical trials are needed.

In cases with a low p-Tyr/m-Tyr+o-Tyr ratio supplementation by p-Tyr could be beneficial, for instance in patients with impaired kidney function, due to chronic kidney disease (Figure 3). This p-Tyr supplementation may compete with the toxic effect of m-Tyr and o-Tyr at the level of protein translation, which should be studied in future human investigations.

GLP-1 receptor agonists are interacting with the intracellular signalling of insulin, leptin, triiodothyronine, acetylcholine and erythropoietin downstream to the IRS, at the Akt, thus they may retain their effectiveness bypassing the critical tyrosine phosphorylation of IRS. Implementation of GLP-1 receptor agonists in a pharmacological dose may cause a break-through of their resistance without causing weight gain (as insulin does), without increasing the number of red blood cells (as erythropoietin is doing), without an elevation of heart rate (which is a characteristic feature of triiodothyronine). Moreover, GLP-1 receptor agonists, in type 2 diabetes, decreased the cardiovascular risk in large randomized, controlled clinical trials [58,59,60,61,62].

In a meta-analysis, the cardiovascular risk reduction by GLP-1 receptor agonists was between 9 and 16% [63], suggesting that their monotherapy is not effective enough, and for that reason, a triple combination of antioxidant, p-Tyr supplementation and GLP-1 receptor agonist may be preferred (Figure 3).

## Figures and Tables

**Table 1:** The sources of para-, meta- and ortho-tyrosine.

Sources of Para-Tyrosine	Sources of Meta- and Ortho-Tyrosine
Food Enzymatic formation Hydroxyl free radical	Food? Enzymatic formation(in plants) Hydroxyl free radical

**Table 2:** Some causes of overproduction and reduced eliminations of abnormal tyrosines.

Overproduction of Abnormal Tyrosines	Reduced Elimination of Abnormal Tyrosines
**Inflammation** *Systemic subclinical e.g.,* Diabetes mellitus Dyslipidaemia *Clinically overt e.g.,* Sepsis	**Chronic kidney disease** End-stage kidney disease Dialysis therapy
